# Differential Modulation of IgT and IgM upon Parasitic, Bacterial, Viral, and Dietary Challenges in a Perciform Fish

**DOI:** 10.3389/fimmu.2016.00637

**Published:** 2016-12-27

**Authors:** Maria C. Piazzon, Jorge Galindo-Villegas, Patricia Pereiro, Itziar Estensoro, Josep A. Calduch-Giner, Eduardo Gómez-Casado, Beatriz Novoa, Victoriano Mulero, Ariadna Sitjà-Bobadilla, Jaume Pérez-Sánchez

**Affiliations:** ^1^Instituto de Acuicultura Torre de la Sal, Consejo Superior de Investigaciones Científicas (IATS-CSIC), Castellón, Spain; ^2^Department of Cell Biology and Histology, Faculty of Biology, Biomedical Research Institute of Murcia (IMIB-Arrixaca-UMU), University of Murcia, Murcia, Spain; ^3^Instituto de Investigaciones Marinas, Consejo Superior de Investigaciones Científicas (IIM-CSIC), Vigo, Spain; ^4^Department of Biotechnology, Instituto Nacional de Investigación y Tecnología Agraria y Alimentaria (INIA), Madrid, Spain

**Keywords:** fish, *Sparus aurata*, immunoglobulins, IgM, IgT, infectious diseases, nutrition, myxozoa

## Abstract

Three different immunoglobulin (Ig) isotypes can be found in teleost fish, IgM, IgD, and the teleost-specific IgT. IgM is considered to have a systemic activity, and IgT is attributed a mucosal role, similar to mammalian IgA. In this study, the complete sequence of gilthead sea bream IgM and IgT in their membrane (m) and soluble (s) forms are described for the first time in a perciform fish. Their constitutive gene expression is analyzed in different tissues, and their regulation upon viral, bacterial, parasitic, mucosal vaccination and dietary challenges are studied. GCB IgM and IgT have the prototypical structure when compared to other fish Igs. The constitutive expression of *sIgM* was the highest overall in all tissues, whereas *mIgT* expression was highest in mucosal tissues, such as gills and intestine. *IgM* and *IgT* were differentially regulated upon infection. *IgT* was highly upregulated locally upon infection with the intestinal parasite *Enteromyxum leei* or systemically after Nodavirus infection. Long-term intestinal parasitic infections increased the serum titer of both isotypes. Mucosal vaccination against *Photobacterium damselae* subsp. *piscicida* finely regulated the Ig response inducing a systemic increase of IgM titers in serum and a local IgT response in skin mucus when animals were exposed to the pathogen by bath challenge. Interestingly, plant-based diets inhibit IgT upregulation upon intestinal parasitic challenge, which was related to a worse disease outcome. All these results corroborate the mucosal role of IgT and emphasize the importance of a finely tuned regulation of Ig isotypes upon infection, which could be of special interest in vaccination studies.

## Introduction

In teleost fish, three different immunoglobulin (Ig) isotypes are found, IgM, IgD, and the teleost-specific IgT which originated from a duplication of the Cμ (1–3). IgT, also named IgZ, was first described in 2005 in rainbow trout (2) and zebrafish (1), hence the two different nomenclatures. The latest trend is to use the term IgT to refer to this teleost-specific isotype. IgT has been initially proposed to have its main role in mucosal immunity, being the functional equivalent in teleosts of mammalian IgA (4, 5), but later studies have shown that the involvement of IgT in the immune response might not be restricted to the mucosal tissues, and thus the humoral response of teleosts is more complex than initially predicted (6). Histochemical studies in rainbow trout intestine revealed that IgM^+^ B cells appear preferentially in the lamina propria and IgT^+^ cells in the epithelium (7), showing a clear physical compartmentalization of these different B cell types. However, the same study showed that oral vaccination against infectious pancreatic necrosis virus (IPNV) induced an increase in both IgT^+^ and IgM^+^ cells, indicating that both cell types are important for mucosal responses. It is widely accepted that IgM expression is dominant in absolute terms in all organs (8–12) and is essential for immune protection against different pathogens upon different routes of infection (1, 9). IgT, despite being generally less abundant than IgM in number of transcripts and cells, is undeniably crucial in mucosal immune responses, but its role in systemic responses (13, 14) and the role of IgM in mucosal responses should not be discarded.

Gilthead sea bream (GSB) (*Sparus aurata*) is a marine species belonging to the Sparidae family (Teleostei: Perciformes). It is the main farmed fish species in the Mediterranean basin (15), representing an important resource for this area. Several diseases hamper their production (16), and therefore, any advancement in the knowledge of its immune response will help to combat diseases. Among these diseases, GSB has been described as a healthy asymptomatic carrier of a highly virulent strain of a Nodavirus responsible for massive mortalities of several species of cultured marine fish (17). No natural GSB mortalities have been associated with this virus strain, and only under experimental conditions, this pathogen can be lethal upon intramuscular injection for small juveniles (18). This role of asymptomatic carrier of a virulent pathogen for other close species such as European sea bass makes this an interesting model to study the immunological response that takes place in GSB hindering the onset of the disease.

The Gram-negative bacteria *Photobacterium damselae* subsp. *piscicida* (*Phdp*) is the causative agent of pasteurellosis, an important disease affecting, among others, juvenile specimens of GSB, up to 100 g, while larger fish are resistant (19). Bath immunization studies of GSB larvae against *Phdp* have shown promising protection results against this disease related to an increase in specific IgM titers (20). Still, under this context, no studies on IgT responses or on the different Ig isotype dynamics have been performed.

*Enteromyxum leei* is an enteric myxozoan parasite that progressively invades the intestinal tract leading to anorexia, reduced growth performance and even death causing important economic loses in Mediterranean sparid farms (21, 22). The slow progression of this disease and the natural niche of the parasite in the paracellular space of the intestinal epithelium make this infection a perfect model to study Ig responses in GSB. More specifically, it constitutes a perfect scenario to investigate the systemic vs. mucosal role for IgT and also IgM. On the other hand, the expanding aquaculture industry has been investigating in the last decade the replacement of fish meal and fish oil (FO) ingredients in aquafeeds by plant proteins and vegetable oils (VO) to increase GSB production (23–25). Among the findings, a diet with a 66% VO replacement showed promising results in growth performance (23). However, upon challenge with *E. leei*, fish fed this VO diet showed a worse disease outcome than those fed FO diet (26). This difference raises questions about the differential dynamics of antibody production and immune responses that different dietary backgrounds can have upon pathological conditions. Previous studies showed that, upon *E. leei* infection, fish fed VO diets had a higher increase in intestinal IgM than FO-fed animals (27), but to date no study has addressed the effect of these alternative diets on IgT and the interactions between the two Ig isotypes.

Many studies have been conducted on fish Ig responses, especially since the relatively recent discovery of IgT (1, 4, 10, 12, 28–33). These studies focused mainly in salmonids and cyprinids and in different fish life stages, upon exposure to different types of pathogens or vaccination strategies, yielding a variety of results that quickly improved the knowledge on this subject. But still, many more studies remain to be performed to unravel the role of the different B cell responses in fish. In the current study, we characterize in detail the soluble (sIg) and membrane (mIg) forms of GSB IgM and IgT, and we analyze their expression dynamics and the different responses upon challenge with several pathogens of very different etiology and infection timing. We complement the study using a mucosal-delivered vaccine and different dietary backgrounds, aiming to decipher the mucosal vs. systemic role of each Ig type under these different scenarios. This integrative study will help to define the dynamics and importance of the different Ig isotypes in teleosts. In addition, considering that most studies about IgT have been performed in cyprinids and salmonids, the current work in a sparid fish will add important information to the knowledge on the evolution of the fish immune system.

## Materials and Methods

### Data Mining for Ig Sequences, Analysis, and Bioinformatics

The sequences of the GSB Igs were identified through data mining of the transcriptomic database of the Nutrigenomics and Fish Growth Endocrinology Group of the Institute of Aquaculture Torre de la Sal^1^ (34). The nucleotide sequences obtained were translated using the ExPASy translate tool,^2^ and specific domains and important sites were predicted using InterPro v.53.0^3^ and VDJ solver 1.0.^4^ N-glycosylation sites were predicted by use of NetNGlyc 1.0.^5^ All alignments were performed with ClustalW v.2.1^6^ and PROMALS3D,^7^ and the phylogenetic tree was constructed using MEGA6 with a neighbor-joining algorithm. Bootstrap values supporting tree nodes derived from 10,000 re-samplings. The variable domains of all Igs were excluded to perform the phylogenetic analysis.

### Fish, Experimental Infections, Feeding Trials Design, and Sampling Procedure

Specific pathogen-free and clinically healthy GSB juvenile specimens from commercial fish farms were kept in 5 μm-filtered and UV irradiated sea water at temperature always above 18°C and below 26°C, with natural photoperiod. Fish were fed *ad libitum* a commercial diet (BioMar, Palencia, Spain) unless otherwise stated. Animals were kept according to the Guidelines of the European Union Council (Directive 2010/63/EU) and the Spanish RD 53/2013.

All efforts were made to minimize suffering of the fish used for the experiments in accordance with national (Royal Decree RD1201/2005, for the protection of animals used in scientific experiments) and the current European Union legislation (86/609/EU), the Bioethical Committees of the University of Murcia for the use of laboratory animals (approval number 537/2011), and the CSIC National Committee on Bioethics under approval number 151-2014.

**Figure 1:**
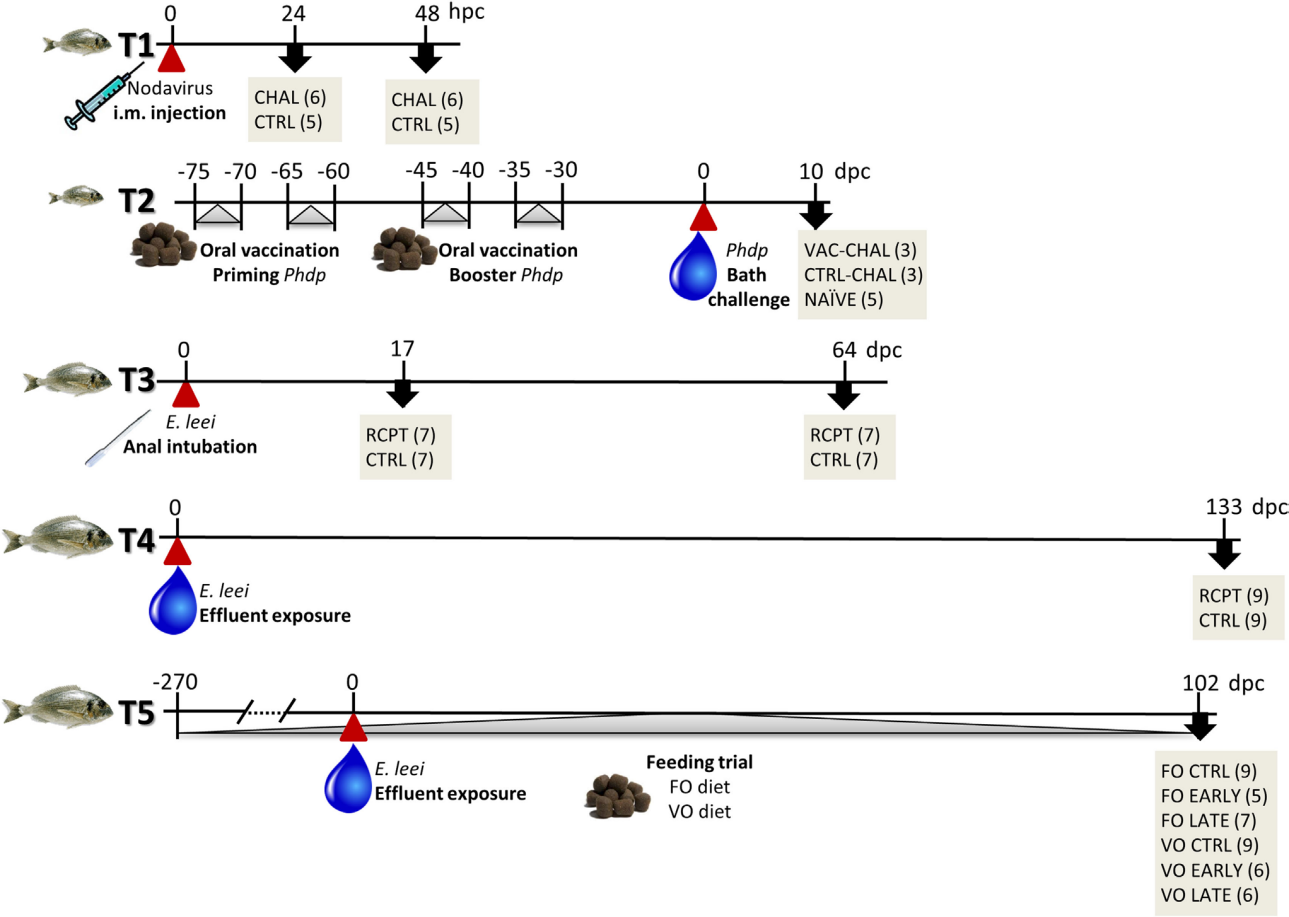
**Schematic representation of the different trials used in this study**. Red arrowheads, always at time-point 0, represent the pathogen challenge with nodavirus (T1), *Photobacterium damselae* subsp. *piscicida* (T2), or *Enteromyxum leei* (T3–T5). Gray arrow heads show intervention periods such as oral vaccination (T2) and dietary challenge (T5). Black arrows point to lethal sampling points including group names and number of fish sampled in gray boxes. More details for each trial are provided in Table 1. dpc, days post-challenge; hpc, hours post-challenge.

To perform this integrative study, five different trials, including challenge with different types of pathogens (virus, bacterium, parasite), immunization, and diets, were performed. For clarity, a summary of the timings and experimental groups can be found in Figure 1, and more experimental details of each trial are summarized in Table 1. Trial 1 (T1) consisted of a viral infection with a nodavirus and was carried out at the experimental facilities of IIM-CSIC (Vigo, Spain). GSB were challenged (CHAL) with the nodavirus strain 475-9/99 (isolated from diseased European sea bass, *Dicentrarchus labrax* L., at the Istituto Zooprofilattico delle Venice, Italy) by intramuscular injection (100 µl of 10^7^ TCID_50_/ml in MEM medium supplemented with penicillin, streptomycin, and 2% fetal bovine serum). The control group (CTRL) was injected with the same volume of the supplemented MEM alone. After 24 and 48 h post-challenge (pc), animals were sacrificed and head kidney and brain tissue samples were collected in Trizol (Gibco) for subsequent RNA extraction.

**Table 1:** **Summary of the experimental conditions of the different trials performed with gilthead sea bream**.

Trial	Pathogen	Type of challenge	Sampling points	Fish weight (g)^a^	*n*_i_^a^	Sampled tissues	Diet
T1	Nodavirus strain 475-9/99	Intramuscular injection	24, 48 hpc	45	30	Head kidney	Basal
						Brain	
T2	*Photobacterium damselae* subsp. *piscidida*	Bath challenge	10 dpc	20	100	Head kidney	Basal + vaccine
						Spleen	
						Intestine	
						Serum	
						Skin mucus	
T3	*Enteromyxum leei*	Anal intubation	17, 64 dpc	60	36	Head kidney	Basal
						Posterior intestine	
T4	*Enteromyxum leei*	Effluent exposure	133 dpc	200	50	Serum	Basal
T5	*Enteromyxum leei*	Effluent exposure	102 dpc	220	60	Head kidney	Fish oil, vegetable oils
						Posterior intestine	

*hpc, hours post-challenge; dpc, days post-challenge*.

*^a^Fish weight and n_i_ refer to the weight and number of fish at the beginning of the trial*.

Trial 2 (T2) was performed at the facilities of the Spanish Oceanographic Institute (Mazarrón, Murcia, Spain) and consisted of an oral vaccination trial against the pathogenic bacterium *Phdp* followed by bath challenge with the same bacterium. GSB were fed at a feeding rate of 1.5% of fish biomass with a commercial pelleted diet (Skretting, Spain), alone or supplemented with an experimental oral vaccine designed for the active immunization of fish against *Phdp*. The vaccine priming was done by administering during 5 days 0.02 ml vaccine per fish plus 0.005 g of repeats of molecular CpG motifs obtained as phenol-extracted genomic *Vibrio anguillarum* DNA (vDNA) (35) as adjuvant, 5 days rest, and again 5 days of treatment to accomplish a total of 0.2 ml vaccine and 50 µg vDNA per fish. Fifteen days later, a booster was performed following the same protocol. For the bacterial bath challenge, the *Phdp* isolate PC.435.1 was prepared as previously reported (20) and animals immersed in the resulting suspension at 10^8^ cfu/ml for 5 h (20). Non-vaccinated fish were kept either non-challenged to serve as a naïve group (NAÏVE) or were challenged to serve as a control (CTRL-CHAL) of the vaccinated and challenged fish (VAC-CHAL). Fish from all the groups were sacrificed 10 dpc for sample collection.

Trials 3 to 5 were performed at the facilities of IATS-CSIC (Castellón, Spain) and consisted of experimental infections with *E. leei* by anal intubation (T3) or by exposure to contaminated effluent mimicking the natural route of infection (T4 and T5). In effluent infections, a group of naïve GSB received water effluent (RCPT) from a donor tank holding *E. leei*-infected GSB, whereas another was kept unexposed (CTRL). In all *E. leei* challenges, in the final lethal sampling, samples were taken to determine the parasite prevalence and intensity of infection by histological analysis of intestines. In T5, additional intermediate non-lethal individual sampling was performed by probing the rectum with a cotton swab, and PCR diagnosis was carried out with primers specific for *E. leei* rDNA as described elsewhere (36).

Trial 3 samples used in the current work come from the infection described in Pérez-Cordón et al. (37). Briefly, GSB were intubated with 0.5 ml of *E. leei-*infected intestinal scrapings (RCPT) or with the same volume of PBS for the control (CTRL) group. At 17 and 64 days post-challenge (dpc), seven fish from each group were sacrificed and pieces of head kidney and posterior intestine were rapidly excised, frozen in liquid nitrogen, and stored at −80°C, until RNA extraction. The prevalence of infection was 42.8 and 85.7% with low and high mean intensities of infection at 17 and 64 dpc, respectively.

Trial 4 samples come from the infection described in Estensoro et al. (27). GSB were set up as effluent RCPT or left unexposed (CTRL). After 133 dpc, the prevalence of infection was 55.6% with a high mean intensity of infection. Serum samples were obtained and kept at −80°C until ELISA was performed. This time-point was chosen based on the findings of Estensoro et al. (27) where the highest IgM transcripts and IgM^+^ cells were found in posterior intestine and head kidney of infected fish.

Trial 5 samples came from the infection described in Estensoro et al. (26). It consisted of a feeding trial combined with an *E. leei* infection. GSB were fed either a FO-based diet or a diet containing a blend of VO at 66% replacement. After the initial feeding period (9 months), fish from both dietary groups were individually tagged with passive integrated transponders and set up as CTRL or effluent RCPT. All fish were non-lethally sampled at 32, 53, and 88 dpc for PCR parasite diagnosis, and classified as early infected (EARLY), when *E. leei*-positive at 32 or 53 dpc, or late infected (LATE), when positive at 88 dpc or later. A final lethal sampling was performed at 102 dpc, and pieces of head kidney and posterior intestine were rapidly excised, frozen in liquid nitrogen, and stored at −80°C until RNA extraction. The final global prevalence of infection was 92.3 and 80% for VO and FO, respectively. Mean intensity of infection was high in EARLY groups, and medium to low in LATE groups, and always higher in VO than in FO fish. All the intestinal samples of RCPT GSB used in the expression analyses of IgT and IgM were selected from *E. leei*-positive fish.

In all the trials, the lethal samplings involved sacrificing the fish by overexposure to the anesthetic MS-222 (Sigma, St. Louis, MO, USA).

### RNA Extraction and Reverse Transcription

Total RNA from the different tissues used in this study, with the exception of T1, was extracted using the MagMAX™-96 total RNA isolation kit (Applied Biosystems, Foster City, CA, USA) following manufacturer’s instructions. Total RNA from head kidney and brain from T1 samples was extracted using Trizol (Gibco, Madison, WI, USA) in combination with the RNeasy Mini Kit (Qiagen, Hilden, Germany) including a DNase treatment with RNase-free DNase set (Qiagen) all following manufacturer’s instructions. The concentration of all RNA samples was determined using a Nanodrop 2000c (Thermo Scientific, Wilmington, DE, USA) and the RNA integrity was assessed on an Agilent 2100 Bioanalyzer. To avoid genomic DNA contamination, 500 ng of RNA samples were treated with DNaseI amplification grade (Invitrogen, Carlsbad, CA, USA), and reverse transcription was performed using the High-Capacity cDNA Archive Kit (Applied Biosystems, Foster City, CA, USA).

### Gene Expression Analysis

Real-time quantitative PCR was carried out with the CFX96 Connect™ Real-Time PCR Detection System (Bio-Rad, Hercules, CA, USA), using a 96-well layout designed for simultaneously profiling all the genes of interest for all individuals studied in each tissue. The primers of the Ig genes studied and the β*-actin*, which was used as a housekeeping gene, are shown in Table 2 and were all checked to have similar efficiencies higher than 90%. All the liquid manipulations required for the PCR were performed by means of the EpMotion 5070 Liquid Handling Robot (Eppendorf, Hamburg, Germany). Each RT reaction of 25 µl contained 660 pg of total input RNA sample, 2× SYBR Green Master Mix (Bio-Rad), and specific primers at a final concentration of 0.9 µM. The PCR reaction was run under the following conditions: an initial denaturation step was carried out at 95°C for 3 min, followed by 40 cycles of denaturation for 15 s at 95°C, and annealing/extension for 60 s at 60°C. The specificity of reactions was verified by analysis of melting curves.

**Table 2:** **Primers used for real-time quantitative PCR**.

Gene name	Symbol	Sequence (5′–3′)	GenBank accession no.
β*-Actin*	*act*β	(F) TCCTGCGGAATCCATGAGA	X89920
		(R) GACGTCGCACTTCATGATGCT	
*IgM* (membrane-bound form)	*mIgM*	(F) GCTATGGAGGCGGAGGAAGATAACA	KX599199
		(R) GCAGAGTGATGAGGAAGAGAAGGATGAA	
*IgM* (secreted form)	*sIgM*	(F) ACCTCAGCGTCCTTCAGTGTTTATGATGCC	JQ811851
		(R) CAGCGTCGTCGTCAACAAGCCAAGC	
*IgT* (membrane-bound form)	*mIgT*	(F) AGACGATGCCAGTGAAGAGGATGAGT	KX599201
		(R) CGAAGGAGGAGGCTGTGGACCA	
*IgT* (secreted form)	*sIgT*	(F) GCTGTCAAGGTGGCCCCAAAAG	KX599200
		(R) CAACATTCATGCGAGTTACCCTTGGC	

### Anti-_GSB_IgT Antibody Production and Validation

A customized polyclonal anti-_GSB_IgT was produced by GenScript (Piscataway, NJ, USA) using as an antigen a selected peptide tested to not cross-react with any other Ig or expressed gene in the GSB transcriptomic database. To verify the specificity of the antibody, 100 µg of skin mucus protein in lysis buffer (20 mM Tris, 0.1 M NaCl, 1 mM EDTA, 0.5% (v/v) Triton X-100, 1 mM PMSF) from RCPT GSB (T4) were resolved in a 4–20% Mini-PROTEAN^®^ TGX™ gel (Bio-Rad) under denaturing [0.1 M dithiothreitol (DTT) and heat treatment 10 min at 95°C] and native (no DTT and heat treatment) conditions, and transferred to nitrocellulose membranes. The membranes were blocked for 2 h with 20 mM Tris 0.5 M NaCl pH 7.4 (TBS) 5% (w/v) non-fat dry milk and incubated overnight with a1:200 dilution of rabbit anti-_GSB_IgT in TBS 1% non-fat dry milk at 4°C. After two 5 min washes with TBS supplemented with 0.05% (v/v) Tween-20 (TTBS) and other two washes with TBS, membranes were incubated with a 1:20,000 dilution of goat anti-rabbit-HRP (Sigma) for 1 h at RT, washed again, and visualized by chemiluminescence detection with Clarity™ Western ECL substrate (Bio-Rad) in an Amersham Imager 600 (GE Healthcare, Little Chalfont, UK).

### Direct and Indirect ELISA Assays

A direct ELISA was used to detect total IgM and IgT in GSB serum (T4). For IgM detection, MaxiSorp 96-well plates (Nunc, Rochester, NY, USA) were coated with 50 µl of a 1:3,000 dilution of fish serum in carbonate/bicarbonate buffer pH 9.6 and incubated overnight at 4°C. After three washes of 5 min with 200 µl TTBS and one 5-min wash with TBS, plates were blocked with 200 µl of TBS 5% (w/v) non-fat dry milk for 2 h at 37°C. Subsequently, the washing steps were repeated and 50 µl of polyclonal rabbit anti-_GSB_IgM (38) 1:40,000 in TBS 3% (w/v) non-fat dry milk were added, and plates were incubated at 37°C for 1 h and washed again. Fifty microliters of goat anti-rabbit-HRP (Sigma) 1:10,000 in TBS 3% non-fat dry milk were added and after 1 h at 37°C and washing, the reaction was developed with TMB peroxidase EIA substrate (Bio-Rad) following manufacturer’s instructions, and the OD at 450 nm was determined with a microplate reader UltraEvolution (Tecan, Männedorf, Switzerland).

To measure total serum IgT, plates were coated with 100 µl of fish serum diluted 1:5 in PBS pH 7.4 overnight at 4°C, washed as described before and blocked with 200 µl PBS 0.05% (v/v) Tween-20 (PBST) 1% (w/v) BSA for 2 h at 37°C. After washing, 100 µl of a customized polyclonal rabbit anti-_GSB_IgT (GenScript) diluted 1:1,000 in PBST 1% BSA were added to the wells, and plates were incubated for 1.5 h at 37°C and washed again. One hundred microliters of goat anti-rabbit-HRP (Sigma) 1:10,000 in PBST 1% BSA were added for 1 h at 37°C, and after the final washes, plates were developed with TMB as described before.

An indirect ELISA was used to assess the specific amount of serum and skin mucus IgT and IgM (T2). Polystyrene microtiter flat wells were sensitized and coated with the antigen, *Phdp* strain PC.435.1. Each assay run included a negative control, a monoclonal antibody against seabream IgM (Aquatic Diagnostics Ltd., Stirling, UK), and a customized polyclonal rabbit anti-_GSB_IgT (GenScript). Both antibodies were diluted 1:1,000 in PBS 1% BSA and further applied following a protocol as previously reported for IgM detection (39). For IgT detection, a slight modification in the secondary antibody was used as described in the previous paragraph. All the steps were carried out at 25°C.

The serum and mucus dilutions used in the different ELISA protocols were previously optimized using a range of serial dilutions of control and experimental samples.

### Statistical Analysis

Statistical analysis was performed using GraphPad PRISM v5.03 and R statistical software v3.0.2 (40). The tests were performed on means after testing that the data met the normality conditions. For normally distributed data, differences were evaluated using the Student’s *t*-test. When conditions were not met, non-parametric tests (Mann–Whitney–Wilcoxon) were used. For multiple comparisons one-way ANOVA followed by the Tukey test or two-way ANOVA followed by the Bonferroni test were used. In all cases, significant differences were considered at *p* < 0.05.

## Results

### Classification of the Three Different Ig Isotypes Found in GSB

**Figure 2:**
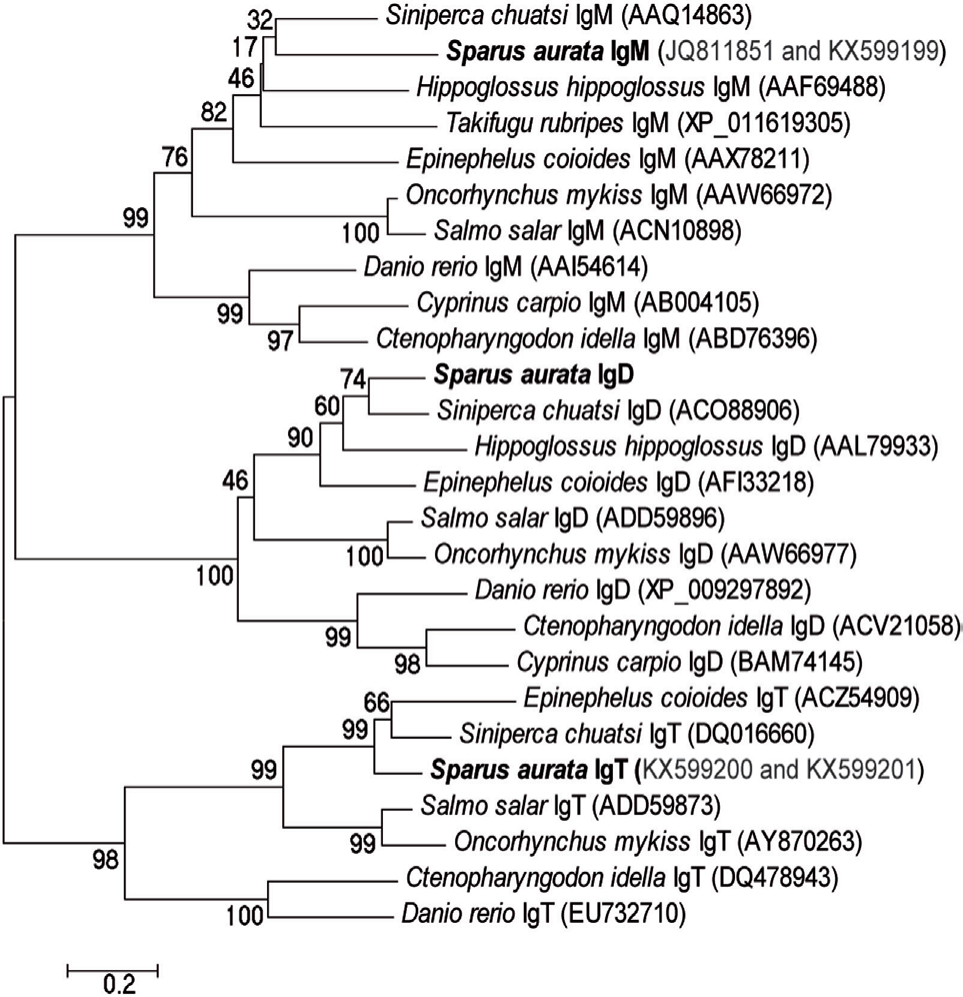
**The three different Ig sequences identified in the database correspond to the prototypical teleost Ig isotyopes**. Phylogenetic tree constructed by aligning the constant domains of the heavy chains of different Ig isotypes (IgM, IgD, and IgT) from different teleost species, including the ones identified in the gilthead sea bream database. The tree was constructed using the neighbor-joining method within the MEGA 6 package and bootstrapped 10,000 times. The GenBank accession numbers of each sequence can be found in parentheses after each species name.

The study of the different Ig isotypes present in GSB was approached by *in silico* analysis of the GSB transcriptomic database. The data mining revealed the presence of full length sequences for both, soluble (s) and membrane (m) bound, heavy chains of IgM and IgT, which were uploaded to GenBank with the following accession numbers: sigm JQ811851, mIgM KX599199, sIgT KX599200, and mIgT KX599201. For IgD, only a partial sequence, comprising three Igδ domains, transmembrane, and intracellular domains, was identified. Phylogenetic analysis, performed by alignment of the constant Ig domains, supported the classification of these three Igs as IgM, IgT, and IgD, as they clustered together with the same isotypes found in different fish species (Figure 2). As expected, IgM and IgD were more closely related than IgT, which grouped apart with high bootstrap values. The three GSB sequences grouped together with the homologous molecule of the closely related mandarin fish (*Siniperca chuatsi*) and the salmonids and cyprinids each grouped in a separate branch within each of the isotypes analyzed, supporting the phylogenetic analysis and evolutionary conservation of these molecules. The subsequent analyses were focused only on GSB IgM and IgT due to the lack of sufficient information in the currently available database to describe the full IgD molecule, and to the complexity of the fish IgD protein.

### GSB IgM and IgT Have the Prototypical Ig Structure

**Figure 3:**
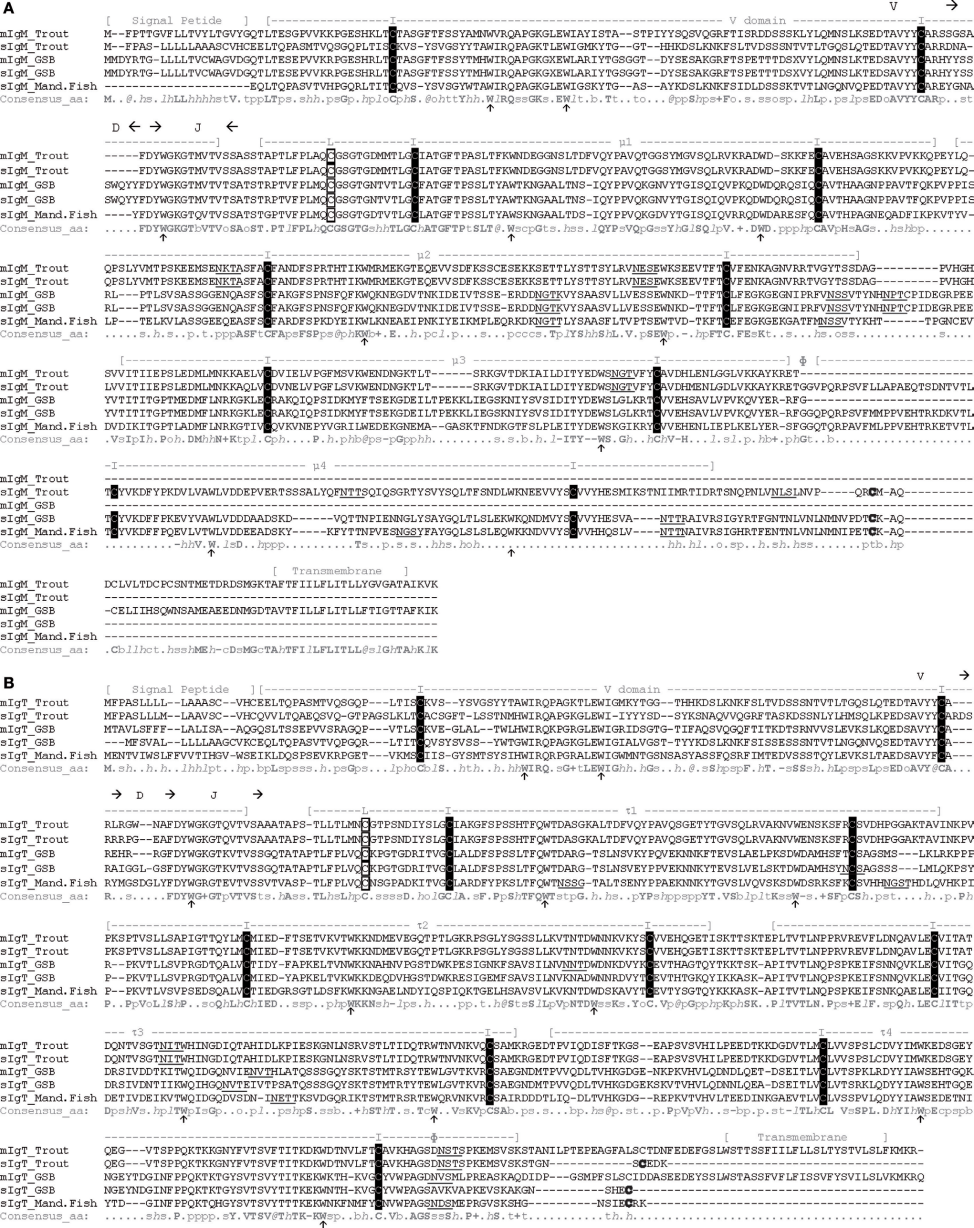
**Gilthead sea bream (GSB) soluble and membrane IgM and IgT are well conserved and have standard residues and structure**. Amino acid sequence alignment of the soluble and membrane forms of IgM **(A)** and IgT **(B)** from rainbow trout, GSB, and mandarin fish (Mand.Fish) performed with PROMALS3D. The signal peptide, transmembrane region, and the stretches of amino acids predicted to constitute the VDJ segment and the variable or the different constant Ig domains (μ and τ) are depicted above the sequences. The alternative splicing region is signaled with Ф above the sequences. Conserved cysteine residues in black squares marked with I are the ones expected to form the intra-chain disulfide bonds, while cysteine residues in white squares marked with L are expected to constitute the disulfide bond to link the light chains to form the complete Ig molecule. Other conserved cysteine residues (bold) can be found at the C-terminal of all soluble Ig molecules. Arrows below the sequence point to the conserved tryptophan residues that help pack the structure. Underlined residues are the predicted N-glycosylation sites. Consensus amino acid (aa) symbols at the bottom of the alignment are: highly conserved aa, bold and uppercase letters; *l*, aliphatic; @, aromatic; *h*, hydrophobic; o, alcohol; p, polar residues; t, tiny; s, small; b, bulky residues; +, positively charged; −, negatively charged; c, charged. The GenBank accession numbers of the sequences used in this alignment are: trout: mIgM AAA56663, sIgM AAW66972, mIgT AAW66979, and sIgT AY870263; mandarin fish: sigm AAQ14863 and sIgT DQ016660; GSB: migm KX599199, sIgM JQ811851, mIgT KX599201, and sIgT KX599200.

The availability of complete transcripts, from start to end codons, of the soluble and membrane-bound forms of IgM and IgT, allowed for the translation of the entire proteins and their sequence analysis. In Figure 3, GSB sequences are aligned with the soluble and membrane forms of rainbow trout (*Oncorhynchus mykiss*) IgT (Figure 3A) and IgM (Figure 3B) and with the mandarin fish sequences. A high degree of conservation can be readily observed. All sequences had a cleavable signal peptide constituted by 17–20 amino acids at the N-terminal, followed by one variable domain, a rearranged VDJ segment, and the constant domains. Both sIgT and mIgT and only sIgM exhibited four constant domains, τ1–τ4 and μ1–μ4, respectively. For mIgM, only three constant domains were present (μ1–μ3). Just after the μ3 domain, the presence of an alternative splicing site allows for the transcription of the transmembrane domain with a very short positively charged intracellular domain of only four amino acids. For IgT, the alternative splicing site was located at the end of the τ4 domain allowing for the insertion of the transmembrane domain without splicing out any of the constant domains. mIgT also had a very short intracellular domain consisting of five amino acids. The predicted molecular weights in dalton were 62.9 for the sIgM, 54.7 for the mIgM, 57.7 for the sIgT, and 63.1 for the mIgT, not taking into account the signal peptide and the putative N-glycosylation sites.

All variable and constant Ig domains can be modeled in the prototypical Ig domain structure (41), consisting of two β-sheets packed face-to-face bound by a disulfide bond formed between two conserved cysteine (C) residues. The presence of the conserved tryptophan (W), which favors the formation and packing of the β-sheet structure, was also detected in all the Ig domains. In μ1 and τ1, an additional conserved cysteine residue was found, which would be expected to form the disulfide bond between these heavy chains and the light chains, forming the complete antibody. The membrane proximal spacer regions of both membrane-bound forms were rich in acid amino acids (D, E) and the transmembrane regions contained residues consistent with the CART domain typical of B cell receptors (42).

### Soluble *IgM* Is the Most Expressed Ig Molecule in All Tissues

**Figure 4:**
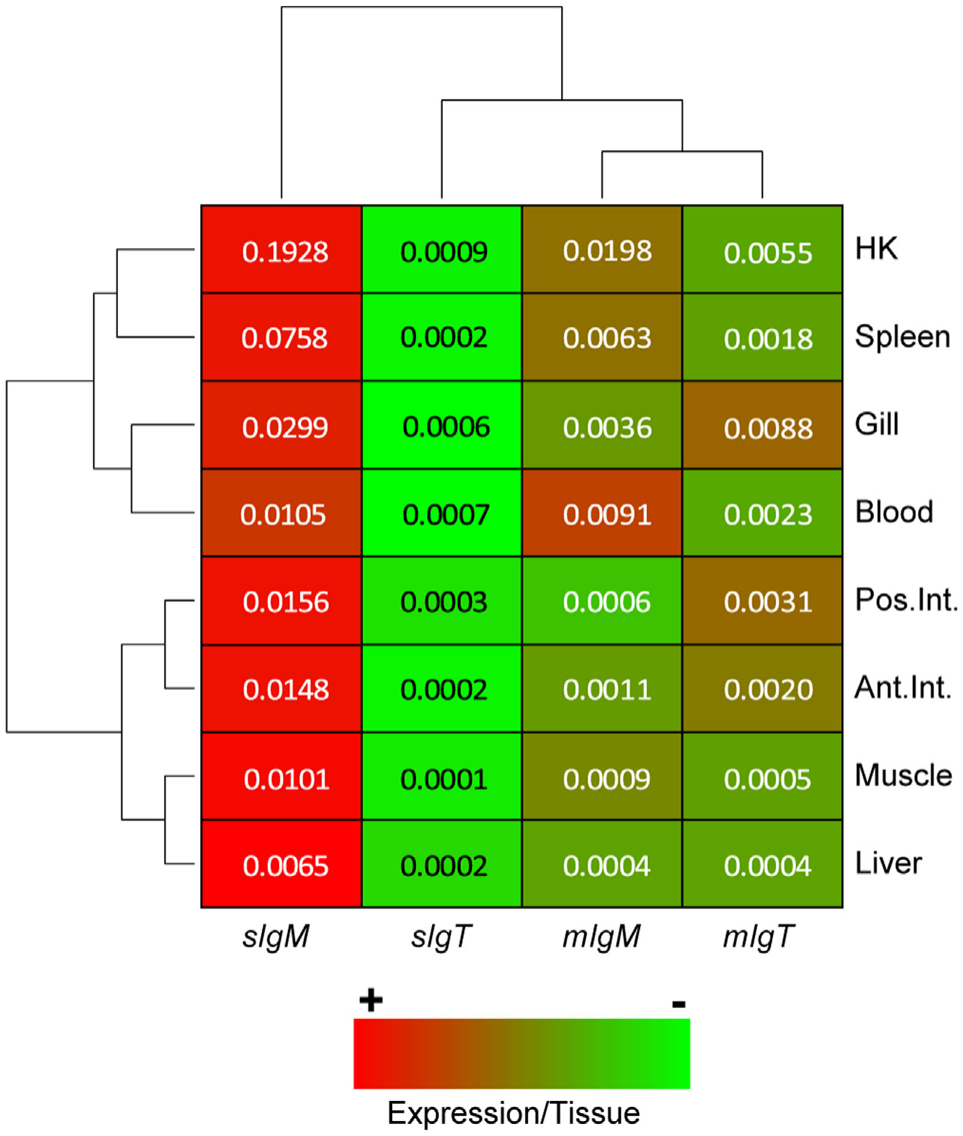
**In basal conditions, *sIgM* is the highest expressed Ig in gilthead sea bream (GSB) organs, while *sIgT* is expressed at extremely low levels**. Heatmap depicting the normalized basal expression of soluble and membrane forms of *IgM* and *IgT* in different GSB organs. Dendrograms show hierarchical clustering of the different Ig molecules and the different tissues based on the Igs expression levels. The color gradient from red to green represents the gradient from high to low values within each tissue. Numbers inside the cells are the basal expression levels calculated using the *β-actin* expression as a reference and are the mean of *n* = 4 GSB.

Once the sequences for the complete soluble and membrane forms of IgM and IgT were known, specific primers were designed and validated to study their basal expression in different tissues of naïve GSB (Figure 4). In all tissues, *sIgM* was the highest expressed Ig and *sIgT* the lowest, being almost undetected in muscle and liver. *mIgM* was highly expressed in blood, almost reaching the levels of *sIgM*. Spleen and head kidney also showed relative high expression levels of *mIgM*, as expected in lymphohematopoietic tissues. In mucosal tissues, such as gill and intestine, *mIgT* showed higher relative expression when compared with *mIgM*, in support of the proposed mucosal role for this Ig.

### Differential Expression Kinetics of *IgT* and *IgM* upon Exposure to Different Pathogens

Three infection models (T1–T3), using viral, bacterial, and parasitic pathogens showed a differential expression of the different forms of *IgM* and *IgT* in lymphohematopoietic organs (head kidney or spleen) and the target organ for each pathogen, brain for nodavirus (18) and intestine for *Phdp* (43) and *E. leei* (44) (Figure 1).

**Figure 5:**
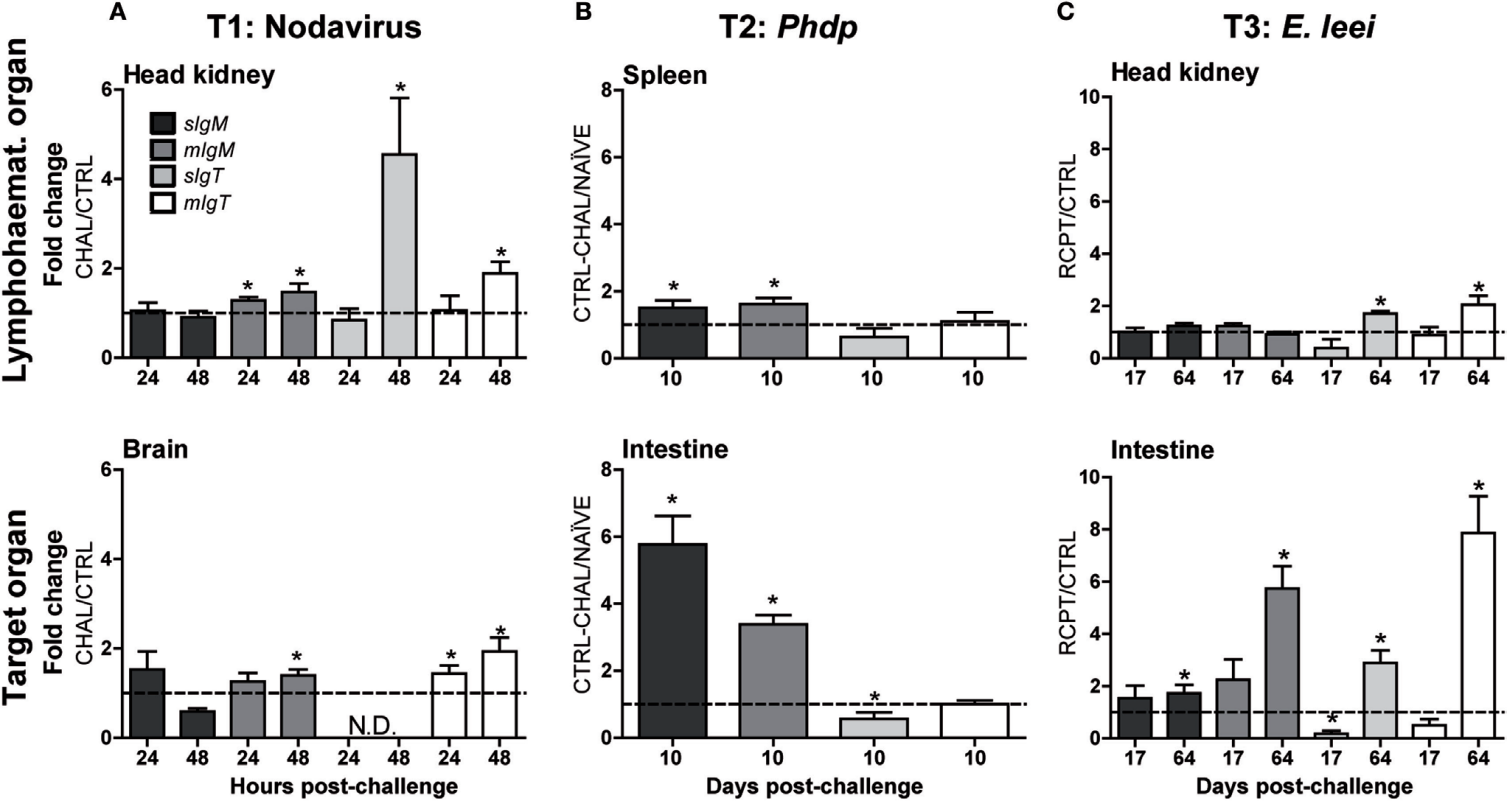
**The different Ig isotypes are differentially regulated upon challenge with different pathogens**. Normalized gene expression analysis of soluble and membrane *IgM* and *IgT* in gilthead sea bream challenged with a virus **(A)** [T1: CTRL vs. challenged (CHAL)], a bacterium **(B)** (T2: NAÏVE vs. CTRL-CHAL), or a parasite **(C)** (T3: CTRL vs. RCPT). The analysis was performed on a lymphohematopoietic organ [head kidney **(A,C)** and spleen **(B)**] and a target organ for the pathogen [brain **(A)** and intestine **(B,C)**]. The gene expression was calculated using the *β-actin* as a reference gene and the expression in non-challenged fish (dotted line at *y* = 1) to calculate the fold changes. Values represent the mean + SE of *n* = 5–6 **(A)**, *n* = 3–5 **(B)**, and *n* = 5–9 **(C)**. Asterisks (*) represent significant differences with the non-challenged group at *p* < 0.05. N.D. stands for not detected.

Gilthead sea bream is an asymptomatic carrier of nodavirus, and the presence of the virus upon intramuscular injection was detected in GSB kidney and brain as fast as 24 h pc (45). In T1, rapid changes in Ig expression were observed (Figure 5A). In head kidney, a slight upregulation of *mIgM* was detected 24 hpc, and both *mIgM* and *mIgT* almost reached a twofold upregulation in head kidney and brain 48 hpc in challenged fish. Of note, whereas *sIgT* was not detected in the brain at any sampling time, a fourfold upregulation was observed in head kidney 48 hpc.

In T2, the bath challenge with *Phdp* did not induce any clinical disease, and all fish successfully cleared the bacteria and survived the challenge. No changes in Ig expression were detected in head kidney (not shown) 10 dpc and only a slight upregulation of both *IgM* forms was observed in spleen of CTRL-CHAL (non-immunized and challenged GSB) when compared to NAÏVE fish (non-immunized and not challenged) (Figure 5B). By contrast, the upregulation of both *IgM* forms was more prominent in the intestine (fourfold to sixfold) than in spleen, but the expression of *IgT* was almost unchanged, with *sIgT* showing a marginal expression decrease when compared with NAÏVE fish.

In the parasite infection trial by anal intubation (T3), the prevalence of infection at 17 and 64 dpc was 42.8 and 85.7% with low and high mean intensity of infection, respectively (37). Almost no differences were observed at the early sampling time (17 dpc) between RCPT (challenged) and CTRL fish (Figure 5C), whereas later on (64 dpc), when the infection was well established, a significant upregulation of all Igs was observed in the intestine of RCPT fish. This upregulation was notably higher for *mIgM* and *mIgT*, suggesting the presence of higher numbers of B cells. In head kidney, only upregulation of both forms of *IgT* was detected.

### IgM and IgT Levels Are Differentially Modulated upon Challenge and Vaccination

**Figure 6:**
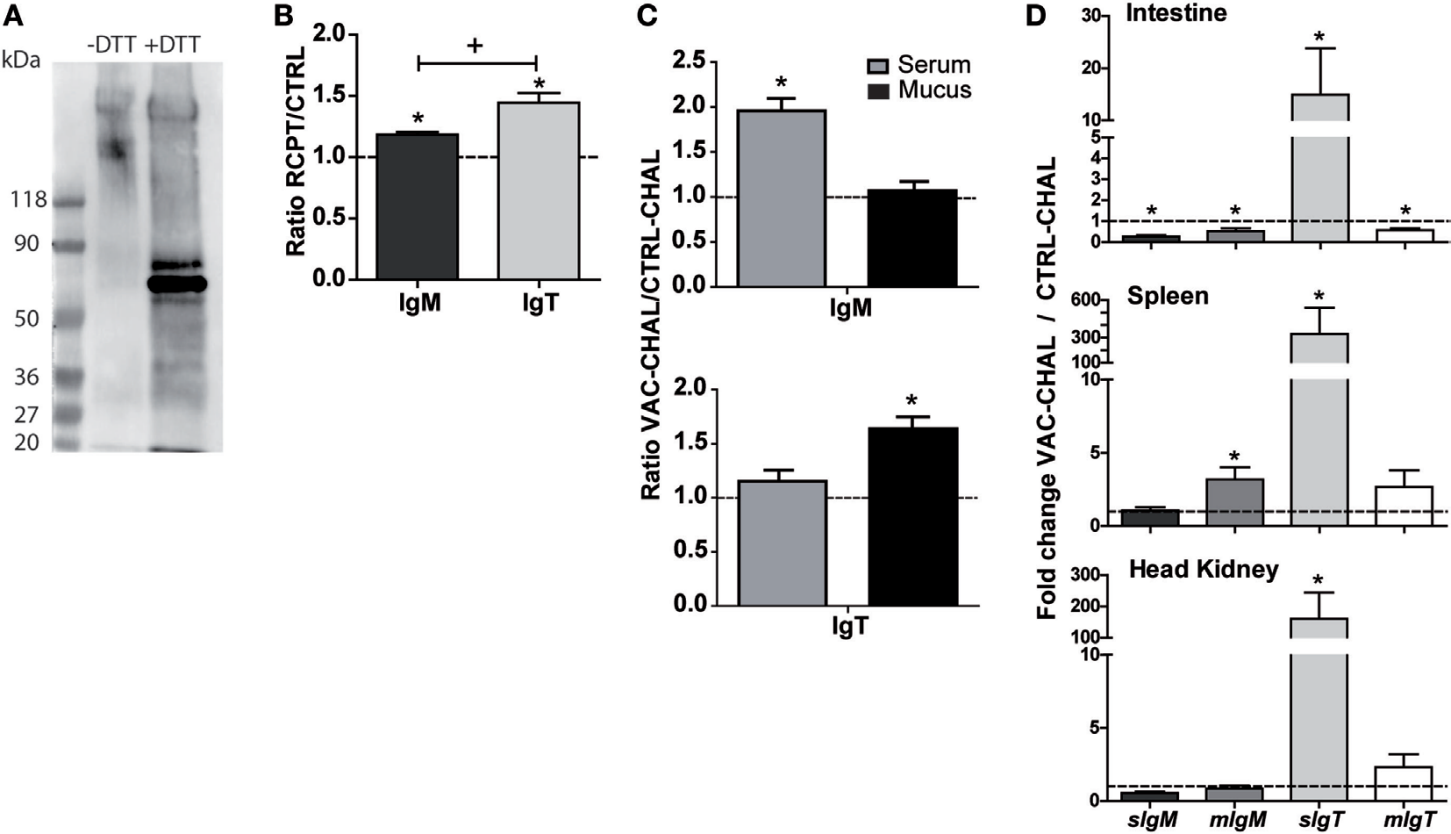
**Differential IgM and IgT secretion can be induced by different challenges and vaccination**. **(A)** Western-blot validation of the anti-_GSB_IgT antibody. Skin mucus samples from RCPT (T4) fish were tested under native and denaturing conditions. The expected molecular weight for sIgT monomers is ~60 kDa. **(B)** Direct ELISA to detect total IgM and IgT in serum samples of CTRL and long-term *Enteromyxum leei*-infected (RCPT) gilthead sea bream (GSB) (T4). Bars show the mean + SE of the ratio between OD RCPT/OD CTRL. *n* = 9 GSB were used for each group. Asterisks (*) represent significant differences with the CTRL fish (dotted line at *y* = 1) and the plus symbol (+) represents significant differences between the ratios IgM–IgT at *p* < 0.05. **(C)** ELISA to detect specific anti-*Photobacterium damselae* subsp. *piscicida* (*Phdp*) IgM or IgT in sera or skin mucus of orally vaccinated [vaccinated and challenged fish (VAC-CHAL)] and control (CTRL-CHAL) fish challenged with *Phdp* (T2). Plates were coated with the antigen used to formulate the vaccine. Data are mean + SE of the ratio between OD VAC-CHAL/OD CTRL-CHAL of *n* = 3 fish. Asterisks (*) represent significant differences between vaccinated (VAC-CHAL) and control (CTRL-CHAL) fish (dotted line at *y* = 1). **(D)** Gene expression analysis of the different Igs in intestine, spleen, and head kidney of the same VAC-CHAL and CTRL-CHAL fish used in **(B)** (T2). Gene expression was normalized relative to the *β-actin* gene and is shown relative to the CTRL-CHAL group (dotted line at *y* = 1). Data are means + SE of *n* = 3 fish. Asterisks (*) indicate significant differences with the CTRL-CHAL samples.

In order to corroborate that the changes in gene expression observed are reflected at a protein level, a customized anti-_GSB_IgT antibody was developed and validated in western-blot, using skin mucus samples from RCPT GSB (T4). The antibody clearly recognized a band of the expected molecular weight for the IgT monomers (~65 kDa) in samples under denaturing conditions. Under non-denaturing conditions, there were no clear bands at the monomer level, whereas bands of higher molecular weight corresponding to IgT polymers were observed (Figure 6A). After validating the antibody, the levels of IgT and IgM in serum and skin mucus samples were studied using ELISA, a technique that allows for quantitative comparisons between different samples.

The effect of the parasite *E. leei* on circulating Igs in fish from a long-term infection (T4) was assessed. Figure 6B shows the ratio of OD values between CTRL and RCPT fish for total IgM and IgT in serum, which resulted in significantly higher values of both IgM and IgT in RCPT than in CTRL fish. Interestingly, the increase in IgT was significantly higher than that in IgM, although IgT values were always lower than IgM ones (IgM OD range 2–2.5, IgT OD range 0.1–0.2; data not shown).

IgT has been proposed to have a more prominent role in mucosal immunity, whereas IgM is known to participate in more systemic responses (4). To study whether this is also the case in our animal model, GSB were orally vaccinated and exposed to the homologous pathogen *Phdp* (T2) and the levels of specific antibodies (IgM and IgT) were measured in serum and skin mucus. After bacterial challenge, the levels of specific IgM antibodies in the serum of vaccinated animals (VAC-CHAL) were significantly increased, remaining unchanged in mucus. On the contrary, the levels of specific IgT antibodies in mucus increased, and no changes were detected in serum (Figure 6C). To support these results, the gene expression of the different Igs of vaccinated fish was analyzed in intestine, spleen, and head kidney and compared to the expression in non-vaccinated fish (CTRL-CHAL). Orally vaccinated fish challenged with *Phdp* showed an increase in expression of *sIgT* in all organs studied, being much higher in spleen. Only *mIgM* was significantly upregulated in spleen, and both *IgM* forms were significantly downregulated in the intestine (Figure 6D).

### Dietary Background Can Cause Inhibition of *sIgT* Expression Leading to a Worse Disease Outcome

**Figure 7:**
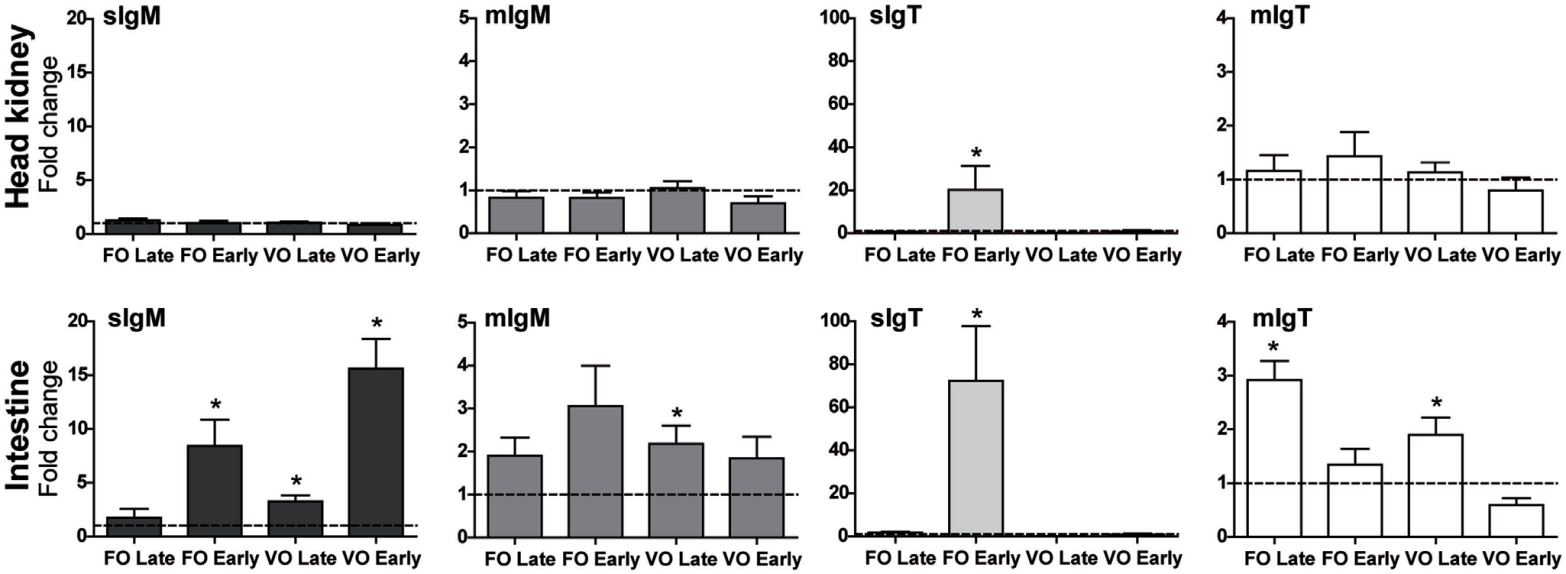
***IgT* expression can be affected by dietary conditions, which can have consequences on disease resistance**. Normalized gene expression analysis of the different Igs in head kidney and posterior intestine of fish fed fish oil (FO)-based normal diets or diets with vegetable oils (VO) at 66% replacement (T5). Fish were tagged individually and kept unexposed (CTRL) or exposed by effluent with the parasite *Enteromyxum leei*. Infected fish were divided in two groups; the group EARLY had a well-established infection while the group LATE had an incipient infection. The gene expression was calculated using the *β-actin* as a reference gene, and the expression in the corresponding VO or FO CTRL groups (dotted line at *y* = 1) was used to calculate the fold changes. Values represent the mean + SE of *n* = 5–9. Asterisks (*) represent significant differences with the FO or VO CTRL group at *p* < 0.05.

Vegetable oil replacement in fish diets has previously shown an effect on disease resistance and immune-related gene expression, including *IgM* (27, 46, 47). *E. leei*-infected GSB fed a VO diet showed an increase in *IgM* transcripts and IgM^+^ cells in the intestine, but exhibited a worse disease outcome (27). This information suggested that VO-fed animals were an interesting model to study whether dietary conditions can locally or systemically affect the Ig response and, subsequently, the capability of the fish to cope with the intestinal infection. To that aim, we analyzed the gene expression of the different Igs in fish fed a control diet (with FO), and fish fed VO replacement diets and subsequently exposed to *E. leei* by effluent for a long period (T5, Table 1). The data on gene expression were grouped according to the infection status of RCPT fish, as some fish get the infection early after exposure (EARLY) and others later on (LATE), and therefore they have well-established or incipient infections, respectively. Ig expression barely changed in the head kidney of any of the groups studied (Figure 7), with the exception of a 20-fold increase in *sIgT* in EARLY fish fed FO. On the other hand, in the intestine, the target of the parasite, several changes were observed. *sIgM* increased expression by 10- to 20-fold in EARLY fish in both dietary groups, with no significant differences between diets. Up to a fourfold increase was also detected in LATE fish fed VO diet, which was also the only group showing a significant increase in the expression of *mIgM*. EARLY fish fed FO diet had a more than 60-fold increase in the expression of *sIgT*, whereas EARLY fish fed VO diet failed to upregulate *sIgT*. The expression of *mIgT* was increased similarly in LATE fish from both dietary groups. No differences were detected when FO and VO CTRL (not challenged) groups were studied (not shown).

## Discussion

Teleost B cells can express three different Ig heavy chains that compose and define the three known Ig isotypes for fish, the evolutionary conserved IgM, IgD, and the teleost-specific IgT (1, 2). IgM^+^/IgD^+^ cells exist in teleost, whereas the expression of IgM and IgT are mutually exclusive (13). The heavy chains of the soluble form of all teleost IgM molecules contain four Cμ domains, while the membrane bound form loses the entire Cμ4 domain during the alternative splicing that allows the insertion of the transmembrane domain (48–50). GSB is no exception and expresses a conserved IgM heavy chain with four Cμ domains in the soluble form and three in the membrane bound form. IgT heavy chains are constituted by four Cτ domains in most fish species (51, 52), but some exceptions exist. As an example, stickleback IgT has three Cτ domains (53), fugu has two (54), common carp can express a chimeric form of IgT which includes a Cμ1 and a Cτ4 domain in addition to the prototypical Cτ1–Cτ4 form (55), and some Antarctic fish can express different IgT transcript variants (56). Within the same species, both mIgT and sIgT have the same number of C domains because the alternative splicing of IgT occurs after the Cτ4 domain (50). The transcriptomic data analysis confirmed that GSB expresses the most common form of IgT with four Cτ domains and, in the case of mIgT, a single transmembrane domain followed by the short cytoplasmic tail. No chimeric or alternative spliced forms were found and, to our opinion, are not expected due to the extensive data contained in the available transcriptome built with information of different tissues, metabolic and immunologic states. Teleosts lack the J chain, important for the Ig polymer’s structural and functional characteristics. However, teleostean IgM and IgT can associate with the secretory component of the polymeric Ig receptor (pIgR) (4, 6). Conserved cysteine residues were found in the C-terminal of all sIgs that can be associated with the secretory pathway. Nonetheless, the prototypical N-glycosylation site and polymerization motif are missing (5) leaving the question of the teleost Ig polymerization mechanism open.

In most teleostean species, the basal expression of *IgM* is dominant, followed by *IgT*. *IgD* expression was always found to be the lowest (10, 11). The highest values of Ig expression are detected in head kidney, generally followed by spleen, in accordance to their established role as lymphohematopoietic organs (57). Our analysis showed that, as expected, in GSB *sIgM* was the most expressed molecule in all studied organs. Plasma cells express extremely high amounts of Igs, therefore, this high expression levels cannot be directly correlated with larger numbers of cells. The expression of *mIgM* was notably higher in blood, being almost the same as the expression of *sIgM*, and this is probably related to the large number of circulating IgM^+^ B cells. The expression of *mIgT* in gill and intestine was higher than that of *mIgM*, confirming the importance of IgT^+^ B cells in mucosal responses (4) also for GSB. The information that can be found in literature concerning basal levels of IgM vs. IgT in mucosal organs has to be interpreted carefully. In Atlantic salmon, *IgM* expression in intestine is higher than *IgT* expression (11); in mandarin fish, more IgM^+^ than IgT^+^ cells can be observed in gills (10); and in rainbow trout IgM is more abundant than IgT in gut and skin mucus (4, 31). Despite these results, it is said that IgT^+^ B cells are the preponderant B cell subset in all mucosal-associated lymphoid tissues (MALT) (58). The interpretation of all these data can be problematic, as most studies on gene expression use primers that do not distinguish between secreted and membrane-bound forms of Igs, and so is the case for the specific antibodies used. The current results help to clarify this, as we show that, although *mIgT* transcripts were more or equally important in mucosal tissues when compared to *mIgM*, the strikingly high basal expression of *sIgM* can mask the importance of IgT, if the secreted vs. membrane distinction is not made.

The first evidence of the mucosal involvement of IgT is very recent. In 2010, Zhang et al. (4) showed that rainbow trout intestinal IgT^+^ cells were the main responders when challenged with an intestinal myxozoan parasite, while IgM responses were only found in serum. In the same study, it was also shown that most intestinal bacteria were mainly coated with IgT. Since then, a plethora of studies have arisen showing the importance of IgT^+^ B cells in MALT. Fish skin, another important mucosal tissue in teleosts, shows an Ig pattern very similar to intestine. IgT^+^ B cells constitute a very important subset of the rainbow trout skin B cell population and IgT is found coating the majority of the skin microbiota specifically responding to skin parasites, whereas IgM changes are only detected in serum (31). Therefore, the compartmentalization of the IgT/IgM responses seems to be indisputable, but the involvement of each isotype is highly dependent on the route of exposure to the pathogen/antigen as illustrated in the following examples. *IgT* gene expression in the gills of grouper (*Epinephelus coioides*) larvae was induced upon bath immunization with a nervous necrosis virus vaccine, whereas in gut, an increase in *IgT* response was only observed upon oral vaccination. By contrast, *IgM* responses were detected systemically upon both immunization routes (33). When rainbow trout were exposed to the viral hemorrhagic septicemia virus (VHSV) by intramuscular injection (13) or were infected by bath challenge (59), IgM and IgT responses were observed in spleen after 3 weeks (13) and in gills only 1 dpc (59), respectively. However, intracoelomic injections (equivalent to intraperitoneal in mammals) with VHSV induced no early upregulation of neither IgM nor IgT in liver (14). Rainbow trout infected with IPNV by bath challenge showed a decrease in serum IgM levels, while no changes were detected in IgT titers (60). Interestingly, intracoelomic injections of rainbow trout with IPNV viral-like particles induced a strong mobilization of IgM^+^ and IgT^+^ cells to the celomic cavity (61). Intracoelomic vaccination of rainbow trout against the bacteria *Yersinia ruckeri* induced *IgM* upregulation, but failed to affect *IgT* expression (62). In general, it is clear that systemic IgM responses are easily elicited by any antigen and route of exposure, whereas IgT modulation mainly occurs when mucosal routes are used or in the presence of pathogens that target specifically mucosal organs and it tends to be restricted to a local environment.

**Figure 8:**
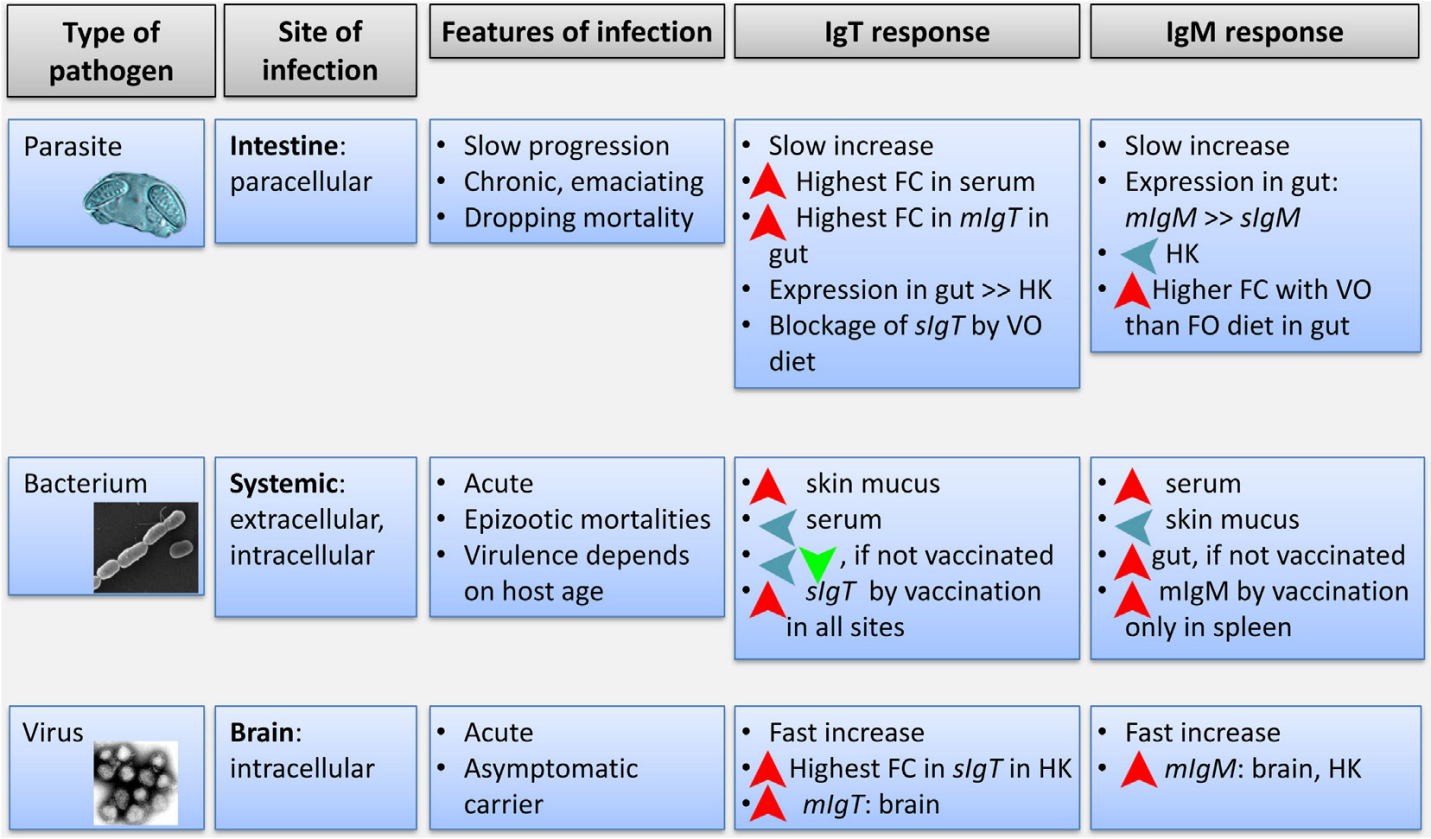
**Integrative diagram of the differential response of IgT and IgM in gilthead sea bream exposed to the three different pathogens used in the study**. Features of each type of pathogen and infection are shown. Fold change refers to changes in gene expression or optical density in ELISA (for serum) in infected fish. HK, head kidney.

Our results, performed in a fish species from a long-distant clade of salmonids, also show that the modulation of the different Ig isotypes depends on the pathogen used and route of exposure. Figure 8 summarizes the different Ig responses to the three pathogen models used in the current study. Nodavirus intramuscular injection induced an increase of *mIgM* in head kidney and brain and a local increase of *mIgT* in brain at early time-points. Previous studies already showed that the immune response at these early time-points was crucial to the resistance of GSB infections with this nodavirus strain. After 24 hpc, nodavirus was already detected in head kidney and brain of GSB and triggered an early and strong pro-inflammatory antiviral response which appears delayed in susceptible species, such as the European sea bass (45). Our results also showed the early triggering of a B cell response, with possible proliferation or migration of IgM^+^ and IgT^+^ B cells in head kidney and brain. No *sIgT* was detected in brain, as expected, as plasma cells are not supposed to be found in normal brain tissue at this early time-point. On the contrary, an increase in *sIgT* was found in head kidney, which possibly means the starting of B cell differentiation in this organ. Although changes in *sIgM* can also be expected, the extremely high basal expression of this molecule can be masking this specific and early response.

The bacterial bath challenge showed the induction of only *sIgM* and *mIgM* in the gut and spleen of naïve GSB. By contrast, when GSB were previously vaccinated against this bacterial pathogen, *Phdp* exposure triggered an extremely important upregulation of the expression of *sIgT* in all the studied organs, while *mIgM* was only upregulated in spleen. No increase in *sIgM* expression was detected in any organ, but again, the high basal expression of this molecule could be masking an increased expression, proliferation, or differentiation of a specific clone/s. For that reason, we found necessary to complement these gene expression results with protein measurement by ELISA. On a protein level, the aforementioned changes in vaccinated animals were reflected in a systemic increase in serum-specific IgM titers and a local increase in skin mucus-specific IgT, which is the first organ in contact with the bacteria upon bath challenge. Therefore, a mucosally delivered vaccine elicited a higher IgM systemic response and a higher IgT local response upon challenge.

Parasites invading mucosal surfaces of rainbow trout [intestine (4) and skin (31)] have been thus far the most informative models for the studies on IgT vs. IgM roles. *E. leei* infections have also been used to study IgM responses in GSB (27). In this long-term infection model, the parasite invades the paracellular space between enterocytes and slowly progresses until it completely occupies the intestinal epithelium, constituting a very interesting model to study the dynamics of IgM and IgT responses. *E. leei* infections are known to induce an increase in *IgM* transcripts (63) and increased numbers of IgM^+^ B cells (27, 64) part of which were proposed to be IgM^+^ plasma cells based on morphological observations. These previous studies also showed that a VO diet induced a further increase in *IgM* transcripts and IgM^+^ B cells after parasite infection. In the current work, experimental *E. leei* infections were performed by two different routes: by directly delivering the parasite to the target organ by anal intubation (achieving faster infections, T3) and by exposure to contaminated water from an infected donor tank (with longer infection times, T4 and T5), which yielded different results. Anal intubation increased Igs expression only after 64 dpc, and this effect was more pronounced in the intestine than in head kidney. Although all the measured Igs were upregulated, the changes in *mIgM* and *mIgT* were more prominent. We propose that this was due to the infiltration or proliferation of intestinal IgM^+^ and IgT^+^ B cells. These findings are in agreement with those found by Estensoro et al. (64) using the same fish–parasite model, where, apart from an increase in IgM^+^ B cells, an increase in IgM^−^ lymphocyte-like cells were observed, which could correspond, at least in part, to IgT^+^ B cells. Regretfully, our anti-_GSB_IgT antibody did not work in immunohistochemistry, so no direct confirmation can be made with the available tools. Infections *via* effluent exposure induced different responses depending on the time of infection establishment. Fish infected for shorter periods (LATE) showed expression patterns similar to those infected by anal intubation. By contrast, fish with well-established infections (EARLY) showed a surprisingly high modulation of *sIgM* and particularly *sIgT*. These changes were also detected in serum, where total titers of IgM and IgT were increased after long-term infections. Although the presence of resident IgT plasma cells in teleost intestines has not been demonstrated so far, our results hint toward an increase in these cells upon long-term parasite exposure. Future efforts should be directed to the development of optimized anti-_GSB_IgT antibodies to better characterize these local IgT responses.

The most interesting finding of this study was the effect a VO diet on *sIgT* expression. Although no changes were observed in fish fed VO diet before parasite exposure, VO diet completely inhibited the upregulation of *sIgT* induced by long-term parasitic infections. We correlate this effect with the worse disease outcome found in fish fed this diet (26). The proposed alleviating role of IgT is in line with the findings in rainbow trout fed certain probiotic diets, which did not show any changes before challenge but, upon cohabitation infections with the bacteria *Lactococcus garvieae*, probiotic-fed fish showed an increased expression of *IgT* in gut, correlated also with a higher protection against infection (65). Of interest, fish fed the VO diet had an increase in intestinal IgM^+^ cells (27) and *IgM* transcripts (current study) upon infection when compared to fish fed the FO diet. This increase appears as an attempt to compensate with IgM the lack of IgT, which seems not to have any positive effect regarding disease outcome, underlining the crucial role of IgT in mucosal responses. These measurements of IgT and IgM show the potential role of these isotypes, but functional evidence for specific protection remains to be demonstrated. In any case, we consider that this model of a dietary intervention that induced a blockade or inhibition of *sIgT* expression is as a very interesting tool to study the role of IgT in intestinal health.

To conclude, the current study is the first to describe the full sequences of the soluble and membrane-bound forms of GSB IgM and IgT and shows for the first time in a fish model their differential expression in different tissues, upon challenge with different pathogens and infection routes. We corroborate the importance of IgT in mucosal responses in a fish species very different to the ones used in most previous IgT studies (salmonids and cyprinids). The current results clearly show that the dynamics of expression of IgM and IgT are very different and depend on the types of pathogen or stimulation, immunization, challenge (intramuscular, anal, bath), tissues, and time after challenge. Finally, we propose the fish–*E. leei* infections and the different dietary interventions as models to further study and unravel the insights of the different Ig isotype functions in teleost fish.

## Author Contributions

AS-B, JP-S, MP, IE, and JC-G conceived and designed the study, analyzed the data, and wrote the manuscript. AS-B, IE, and MP performed the parasite challenges, sample manipulation, and assays associated with it, including ELISA and western-blot. JP-S and JC-G performed all gene expression experiments and analyses in the study. JG-V and VM conceived and performed the bacterial vaccination/challenge experiment and sampling and performed specific ELISA assays. BN and PP performed the viral challenge, sampling, and RNA extraction of such samples. EG-C carried out the epitope study for the antibody development. All the authors reviewed the manuscript.

## Conflict of Interest Statement

The authors declare that the research was conducted in the absence of any commercial or financial relationships that could be construed as a potential conflict of interest.

## Footnotes

^1^http://nutrigroup-iats.org/.

^2^http://web.expasy.org/translate/.

^3^http://www.ebi.ac.uk/interpro/.

^4^http://www.cbs.dtu.dk/services/VDJsolver/.

^5^http://www.cbs.dtu.dk/services/NetNGlyc/.

^6^http://www.genome.jp/tools/clustalw/.

^7^http://prodata.swmed.edu/promals3d/promals3d.php.

## Abbreviations

GSB, gilthead sea bream; sIg, soluble immunoglobulin; mIg, membrane-bound immunoglobulin; *Phdp, Photobacterium damselae* subsp. *piscicida*; CTRL, control; RCPT, recipient; VAC, vaccinated; CHAL, challenged; pc, post-challenge.
